# Lipophilic Micronutrients and Adipose Tissue Biology

**DOI:** 10.3390/nu4111622

**Published:** 2012-11-06

**Authors:** Jean-François Landrier, Julie Marcotorchino, Franck Tourniaire

**Affiliations:** 1 Institut National de Recherche Agronomique (INRA), UMR 1260, F-13385, Marseille, France; Email: julie.marcotorchino@univ-amu.fr (J.M.); franck.tourniaire@univ-amu.fr (F.T.); 2 Institut National de la Santé et de la Recherche Médicale (INSERM), Nutrition, Obésité et Risque Thrombotique, UMR 1062, F-13385, Marseille, France; 3 School of Medicine, Aix-Marseille University, F-13385, Marseille, France

**Keywords:** adipose tissue, adipocytes, vitamins, micronutrients, obesity, insulin resistance, inflammation, metabolism, adipogenesis

## Abstract

Lipophilic micronutrients (LM) constitute a large family of molecules including several vitamins (A, D, E, K) and carotenoids. Their ability to regulate gene expression is becoming increasingly clear and constitutes an important part of nutrigenomics. Interestingly, adipose tissue is not only a main storage site for these molecules within the body, but it is also subjected to the regulatory effects of LM. Indeed, several gene regulations have been described in adipose tissue that could strongly impact its biology with respect to the modulation of adipogenesis, inflammatory status, or energy homeostasis and metabolism, among others. The repercussions in terms of health effects of such regulations in the context of obesity and associated pathologies represent an exciting and emerging field of research. The present review will focus on the regulatory effects of vitamin A, D, E and K as well as carotenoids on adipose tissue biology and physiology, notably in the context of obesity and associated disorders.

## 1. Adipose Tissue, an Endocrine Tissue Involved in Obesity and Associated Pathologies

Adipose tissue is an organ actively involved in maintaining metabolic homeostasis. White adipose tissue (WAT) was initially regarded as a protective and supportive tissue allowing the storage of excess energy as triglycerides (lipogenesis) and the release of energy as fatty acids (lipolysis). Brown adipose tissue (BAT) is mainly involved in the control of thermogenesis. Adipose tissue is now also regarded as an endocrine tissue producing not only free fatty acids but also a large variety of hormones, cytokines, chemokines and growth factors acting on metabolism, vascular and endothelial functions, appetite and satiety, immunity, fertility, inflammation and many other physiological processes. The complex and tightly regulated process of adipocyte development is called adipogenesis. This process has been intensely studied, and the temporal sequences as well as transcriptional regulators involved have been identified. Among them, the nuclear receptor peroxisome proliferator-activated receptor gamma (PPARγ) and the CCAAT-enhancer-binding protein (CEBPs) families are considered as transcriptional regulators of adipogenesis [1]. 

Obesity is characterized by an excess of fat mass corresponding to the expansion of adipose tissue linked to hypertrophia and/or hyperplasia of adipocytes [2]. A new concept of obesity has also emerged, defining obesity as a disease associated with chronic low grade inflammation characterized by abnormal secretion of cytokines, acute phase proteins and other mediators of the immune response together with the activation of inflammatory signaling pathways [3,4]. 

Adipose tissue is a major contributor to the chronic inflammatory response. The origin of the regulation of molecules secreted by adipose tissue is multifactorial and is linked to several physiopathological disorders, including: (1) increased levels of circulating free fatty acids, (2) hypoxia of hypertrophied adipose tissue, (3) systemic and local oxidative stress, (4) endoplasmic reticulum stress and/or (5) the production of inflammatory cytokines. All these types of stress converge towards signaling pathways involving c-Jun amino-terminal kinase (JNK) and IκB kinase β (IKKβ), which are central to obesity-associated insulin resistance [3,4].

Adipose tissue-secreted compounds include free fatty acids and approximately 50 biologically active proteins grouped under the term “adipokines”. Adipokines act in an autocrine, paracrine and/or endocrine fashion. Adipocytes used to be considered the main source of adipokines, but recent studies have found that a large subset of them are actually produced by cells belonging to the stromal vascular fraction of adipose tissue, especially macrophages. The number of infiltrated macrophages increases proportionally to the expansion of adipose tissue, and it has been positively correlated with adiposity, adipocyte size and insulin resistance [5]. Macrophages also participate in adipose tissue function/dysfunction. They interfere with adipocyte function through the production of pro-inflammatory cytokines such as TNF-α, IL-1β and IL-6, which can lead to insulin resistance, modify adipokine secretion and lead to an excess of free fatty acid secretion through increased lipolysis and diminished lipogenesis [6]. 

The main molecules secreted by adipose tissue and involved in inflammation and the development of insulin resistance are:

- Free fatty acids: continuously released from adipose tissue, with a peak in secretion during fasting and a decrease during postprandial periods. During obesity, when resistance of adipose tissue to insulin develops (partly because of hypoxia generated following adipocyte hypertrophia/hyperplasia), enhanced lipolysis leads to a massive increase in plasma free fatty acids. Free fatty acids will then perturb liver and muscle insulin action.
- Adiponectin: mainly synthesized by adipocytes [7,8]; elevated circulating concentrations are found in the plasma of lean individuals (5–30 mg/L). Conversely to other adipokines, its production and secretion are diminished in insulin resistant or Type 2 diabetic obese individuals. Adiponectin increases insulin sensitivity and modulates hepatic glucose synthesis by inhibiting the expression of enzymes essential to gluconeogenesis. Adiponectin also presents anti-inflammatory properties through its ability to modulate the expression of pro- and anti-inflammatory cytokines (especially TNF-α).
- Leptin: similarly to adiponectin, leptin is mainly produced by adipocytes [9]. Circulating levels of leptin as well as its expression in adipose tissue are positively correlated with the severity of obesity [10]. Leptin’s main action is on the central nervous system to regulate food intake. Furthermore, leptin possesses pro-inflammatory properties: It stimulates the production of TNF-α, IL-6 and IL-12 by macrophages. Conversely, it also improves insulin sensitivity by activating AMP-activated protein kinase (AMPK). However, obese individuals display very high circulating levels of leptin, suggesting the existence of leptin resistance in these subjects [10].
- Pro-inflammatory cytokines: TNF-α, IL-6 and IL-1β are synthesized by numerous tissues, including obese adipose tissue, which, in addition to their well described pro-inflammatory properties, are involved in the genesis of insulin resistance. TNF-α was the first pro-inflammatory mediator linked to inflammation, obesity and insulin resistance [11]. TNF-α and IL-1β interfere with insulin signaling at the level of insulin receptor substrate-1 (IRS-1) in different ways, whereas the mechanisms of action of IL-6 are still a matter of debate [10,12].
- Plasma retinol binding protein 4 (RBP4), produced mainly by the liver, has also been shown to be an adipokine, favoring insulin resistance in mice [13]. However, clinical observations did not show a consistent correlation between plasma RBP4 and obesity associated insulin resistance, but this lack of a correlation may be caused by confounding factors, including methodology [14]. Several authors have therefore proposed to use the retinol:RBP4 ratio rather than RBP4 levels alone as a better marker (a low retinol:RBP4 is associated with insulin resistance), suggesting that retinol-free RBP4 (apo-RBP4) might contribute more to insulin resistance than retinol-bound RBP4 (holo-RBP4) [15,16]. Recently, two mechanisms by which RBP4 induces insulin resistance have been proposed, suggesting that both apo- and holo-RBP4 could participate in tissue insulin resistance. In a first report, it was shown that binding of holo-RBP4 to its membrane receptor, sensitive to retinoic acid-6 (STRA6, mediating cellular uptake of retinol), triggers an inflammatory signaling cascade (janus activated kinase 2/signal transducer and activator of transcription 5, JAK2/STAT5), causing a blockade of insulin response via the induction of expression of suppressor of cytokine signaling-3 (SOCS3), but also an increase in PPARγ, which stimulates lipid accumulation [17]. On the other hand, Norseen *et al.* have shown that insulin resistance could be triggered indirectly in adipocytes by either apo- and holo-RBP4 [18]. Indeed, both forms of RBP4 could stimulate pro-inflammatory cytokine secretion by macrophages via JNK and TLR4 pathways, independently of STRA6 binding, hereby perturbating insulin pathways in adipocytes.
- Chemokines: adipocytes secrete several chemoattractive molecules [19], in particular for macrophages. Monocyte chemoattractive protein-1 (MCP-1), the secretion of which is dramatically increased in obese adipose tissue, is a key mediator for the recruitment of macrophages in adipose tissue [20].

## 2. Impact of LM on Obesity and Associated Pathologies

Obesity and associated disorders, such as low-grade inflammation or insulin resistance, have been associated in many epidemiological and observational studies with low circulating concentrations of lipophilic micronutrients (LM) or, in some cases, to low LM concentrations in adipose tissue. Indeed, deficiencies in LM are often documented in obese people [21,22]. In addition, many obesity-associated disorders are also strongly inversely associated with serum LM concentrations [23,24,25]. 

Due to the well-established role of LM in gene expression via many molecular mechanisms, including highly specific nuclear receptors in the case of vitamin A and D metabolites or less specific signaling pathways such as mitogen activated protein (MAP) kinases or nuclear factor κB (NF-κB) in the case of carotenoids and retinoic acid (as described below for each LM), it is tempting to hypothesize that LM could directly modulate gene expression in adipose tissue, and consequently affect adipose tissue biology. In this way, LM may thus prevent or at least limit obesity and associated disorders. This assumption was motivated by (1) the ability of LM and metabolites to regulate gene expression, (2) the key role of adipose tissue in obesity and associated disorders, (3) modulation of adipogenesis, inflammation or metabolism gene expression in adipose tissue having systemic physiological consequences, (4) the expression and function of signaling pathways and nuclear receptors in adipose tissue and in adipocytes and (5) the fact that adipocytes constitute one of the main reservoirs within the body for LM, which renders the direct molecular effects of LM on adipocytes feasible. All of these effects of LM on adipocytes/adipose tissue biology will now be described. 

## 3. Vitamin A

**Figure 1:**
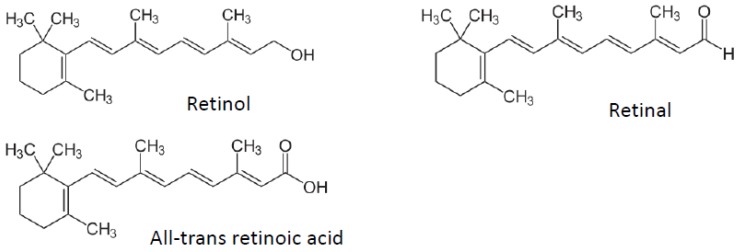
Vitamin A and metabolite structures.

Vitamin A (Figure 1) can be found in four main forms within the body: retinol (circulating form), retinyl-esters (storage form), retinal and retinoic acid (which represents its most active form). Retinol can be converted reversibly to retinal by alcohol dehydrogenases (ADHs) and retinal can be irreversibly converted to retinoic acid by retinaldehydrogenases (RALDHs). Retinol is also stored in tissues after esterification with fatty acids as retinyl esters by lecithin-retinol acyltransferase and acyl CoA:retinol acyltransferase. Finally, retinoic acid is inactivated by catabolism by cytochrome P450 hydroxylase 26A and glutathione *S*-transferase enzymes (for an extensive review on vitamin A metabolism in the body, see d’Ambrosio *et al.* [26]).

Adipose tissue is the second most important storage site for vitamin A after the liver. Tsutsumi *et al.* estimated that it represents 15%–20% of the total body store in rats [27]. They also found that vitamin A is mostly found as free retinol in the adipocyte fraction of adipose tissue, with almost no retinol present in stromal vascular cells. Moreover, adipose tissue expresses all the enzymes necessary for vitamin A transport (including intracellular binding proteins) and metabolism. Several isomers of retinol, including all-trans, 9-*cis* and 13-*cis* isomers, have been quantified in WAT. Furthermore, retinol content was found to be similar among the different depots (*i.e.*, visceral and subcutaneous) and in brown and white tissues [27,28,29]. Retinal has also been found in the adipose tissue of mice [30], as have several isomers of retinoic acid, except 9-*cis* retinoic acid [31,32]. Despite being regarded as the natural ligand for retinoid X receptors (see below) *in vitro*, 9-*cis* retinoic acid is extremely difficult to detect *in vivo* because of its lability and because it most likely occurs at very low concentrations or in a specific cell population within tissues [33]. However, it should be noted that 9-*cis* retinol is present in WAT [28] and that WAT expresses RALDHs [29], therefore 9-*cis* retinoic acid could be produced in adipose tissue.

Two families of receptors (retinoic acid receptors, RARs, and retinoid X receptors, RXRs) mediate the effects of retinoids [34,35]. Three subtypes of each have been described (RARα RARβ, RARγ, RXRα, RXRβ and RXRγ). These receptors work as ligand-dependent transcriptional regulators by binding specific DNA sequences [Retinoic Acid Response Element (RARE) or Retinoid X Response Element (RXRE)] found in the promoter region of retinoid target genes either as RAR-RXR or RXR-RXR dimers. All-trans retinoic acid (ATRA) can bind RARs only, whereas 9-*cis* retinoic acid is a ligand for both. RARs and RXRs subtypes are found in every cell type. Furthermore, RXRs are dimerisation partners for other nuclear receptors such as peroxisome proliferator activated receptors (PPARs), liver X receptor (LXR), farnesoid X receptor (FXR) and pregnane X receptor (PXR), RARs, thyroid hormone receptor (TR) and vitamin D receptor (VDR). These characteristics explain why retinoic acid is involved in the regulation of expression of several hundred genes. In addition several other transcription factors and signaling pathways are modulated by retinoic acid [36]. 

Interestingly, the expression patterns of RARs and RXRs differ between brown and white adipose depots, with RARα, RARγ and RXRα expressed more in WAT, and RARβ and RXRγ expressed more in BAT [37,38,39], suggesting different gene expression regulation patterns in response to retinoic acid treatment.

### 3.1. Adipogenesis

Whereas Safonova *et al.* (1994) showed that very low concentrations of ATRA (in the nM range) promoted adipogenesis *in vitro*; other studies using retinoic acid concentrations in the µM range have shown an anti-adipogenic effect [40]. Murray and Russell first showed in 1980 that retinoic acid was able to block the differentiation of pre-adipocytes into adipocytes *in vitro*, but did not interfere with cell proliferation [41]. This result was confirmed in other cell lines [42,43,44,45,46,47,48,49]. Further studies have specified the periods during which retinoic acid could prevent adipocyte differentiation during adipogenesis [50]. It was then established that retinoic acid blocks the differentiation process by inhibiting the transcriptional activity of CEBPβ, resulting in the blockade of PPARγ, the master regulator of adipocyte differentiation [1,51]. More recent work indicated that retinoic acid acts upstream by inducing the expression of Pref-1, an inhibitor of adipocyte differentiation solely expressed in pre-adipocytes, Sox9 and KLF2, resulting in inhibition of the expression of the adipogenic proteins CEBP and PPARγ, and sterol responsive element binding protein 1c (SREBP1c) [52].

### 3.2. Inflammation

Despite being known as a major regulator of immune response [53], only a few studies have shown that vitamin A has positive effects by decreasing the expression of inflammatory mediators by adipocytes including adipsin [54] and resistin [55]. Our group has also shown that ATRA is able to limit cytokine expression in TNF-α-treated 3T3-L1 cells [56]. 

### 3.3. Metabolism

The absence of vitamin A in the diet produces growth retardation but increases adiposity [57], which is characterized by increased adipocyte size, possibly caused by increased expression of PPARγ in WAT [58]. On the other hand, supplementation studies with retinol or retinoic acid have shown that vitamin A is able to reduce fat mass and improve insulin sensitivity in rodents. The molecular mechanisms underlying these changes have been investigated *in vitro*. Retinoic acid-treated adipocytes show a decrease in triglyceride content and an increase in lipid oxidation, which was related to the overexpression of lipid metabolism genes such as acyl-CoA oxidase (ACO) and carnitine palmitoyltransferase 1-L (CPT1-L) [59]. It is noteworthy that opposite effects have also been reported [60].

Vitamin A supplementation studies have been performed in lean animals fed a standard (*i.e.*, non-obesogenic) diet. Palou and co-workers have highlighted the fact that acute treatment (4 days) of mice with retinoic acid leads to significant weight loss and a decrease in WAT mass, characterized at the tissue level by a smaller adipocyte size [61,62,63]. Retinoic acid administration also prevented weight gain and improved insulin sensitivity in genetically obese ob/ob mice [64]. Vitamin A supplementation in the diet of genetically obese WNIN/Ob rats resulted in a decreased total body weight related to a decrease in adiposity and lowered glycemia and insulinemia [65,66]. Interestingly, similar results were reported using either all-*trans* or 13-*cis* RA [67]. Sakamuri *et al.* attributed this anti-obesity effect to the ability of vitamin A to decrease the activity of the 11β-hydroxysteroid dehydrogenase type 1 (11β-HSD1), an enzyme involved in the synthesis of glucocorticoids [68], hormones that have been shown to promote pre-adipocyte differentiation [69]. This effect was explained by the fact that 11β-HSD1 gene expression is controlled by CEBPα, a downstream target of PPARγ involved in terminal adipocyte differentiation [68]. Therefore, decreased glucocorticoid synthesis might be another mechanism by which vitamin A modulates adiposity.

Other studies have investigated the influence of vitamin A supplementation on diet-induced obesity. Felipe *et al.* found no effect on weight gain or adiposity in mice fed a high fat diet supplemented with vitamin A [70]. Similarly, Bairras *et al.* fed rats with a cafeteria diet containing either a normal amount or a supraphysiological amount of vitamin A [71]. Despite a decrease in food intake and an increase in adipose PPARγ and RXRα, they found no significant improvement in adiposity or body weight between the two groups. On the other hand, retinoic acid could limit the adverse consequences of a high fat diet in terms of weight gain, adiposity and blood lipids. The discrepancy in the effects could arise from the fact that vitamin A was supplied to the animals in the form of retinyl-esters in the food, whereas Berry and Noy used retinoic acid and/or retinoic acid delivered by a subcutaneously implanted retinoic acid pellet, therefore avoiding absorption and metabolism of the retinyl-esters [72]. In addition, Berry and Noy showed that anti-obesity and insulin sensitive properties are mediated, at least partially, by PPARδ, for which ATRA has been previously described to be a ligand [73]. The binding of ATRA to RAR or PPARδ appears to be dependent on the expression level of the retinoic acid intracellular binding protein cellular retinol binding protein 2 (CRBP2) and fatty acid binding protein 5 (FABP5). Upon binding to CRPB2, the RAR signaling pathway is activated, while binding to FABP5 induces expression of *PPARδ* target genes [72].

Interestingly, there is evidence that another metabolite of retinol, retinal, also plays a role in the regulation of fat mass in mammals. Ziouzenkova *et al.* showed that retinal is able to block 3T3-L1 differentiation and that retinal content is decreased in the adipose tissue of high fat diet fed mice [30]. Moreover, Raldh1-null mice, which accumulate retinal in their adipose tissue, are resistant to diet-induced obesity, display smaller adipocyte size and a better insulin response and lipid profile than their wild-type counterparts [30]. Raldh1^−/−^ adipocytes also tend to acquire features of BAT [74]. In line with these observations, retinol deshydrogenase-1^−/−^ (Rdh1) mice, which lack the main isoform of the enzyme necessary for retinal synthesis from retinol, become fatter than wild-type animals when fed a vitamin A deficient diet [75]. Interestingly, Rdh1 is not expressed in WAT, indicating that vitamin A metabolism can also indirectly influence adipose tissue biology. Furthermore, retinal has been shown to decrease RBP4 expression in murine embryonic fibroblast (MEF) cells [76]. 

Recently, a retinol saturase (RetSat) has been identified in mammals [77]. RetSat catalyzes the synthesis of ATRA to all-*trans*-13,14-dihydroretinol, which can be further converted to all-*trans*-13,14-dihydroretinoic acid, which is an RAR ligand [78,79], but that has a lower transactivation efficiency compared with ATRA *in vivo*. Interestingly, RetSat is necessary for 3T3-L1 adipocyte differentiation, and the expression level of RetSat is diminished in the adipose tissue of obese individuals [80], while RetSat knock-out animals have increased adiposity [81].

To explain the anti-obesity effects of vitamin A, much attention has been paid to its ability to induce uncoupling proteins (UCPs) in both BAT and WAT. UCPs are mitochondrial proteins that uncouple respiration from ATP production, resulting in heat production [82]. Early works by Alvarez *et al.* and Puigserver *et al.* showed that 9-*cis*-retinoic acid and ATRA are able to induce UCP1 in BAT *in vitro* and *in vivo * [83]. This effect was mediated by RARs and RXRs [84] but also by p38 MAPK [85]. At this time, only a single isoform of UCP was known, but subsequent research led to the identification of two additional isoforms, UCP2 and UCP3. Interestingly, retinoic acid was able to induce the expression of UCP1 and UCP2 but not UCP3 in brown and white depots *in vitro* [55,63,86,87,88,89] and *in vivo* [70]. On the other hand, UCP1 protein content is decreased in the BAT of mice fed a vitamin A deficient diet [90]. 

The increase in fatty acid oxidation and energy dissipation via UCP1 is largely mediated by the activation of the PPARγ co-activator-1 (PPARGC1A), a transcriptional coactivator of PPARγ, and nuclear respiratory factors 1 and 2 (NRF1 and NRF2), leading to increased mitochondrial function and activity [83]. PPARα, which regulates the expression of genes involved in fatty acid oxidation, is also induced in response to retinoic acid treatment [91].

Another way vitamin A can improve the metabolic profile is through the modulation of circulating leptin levels. Several studies have described an inhibitory effect of retinoic acid on leptin expression. Vitamin A isomers have been shown to diminish the expression of leptin in adipose tissue explants but also *in vivo* in vitamin A supplemented rats [87,88,92] and mice [62,70], leading to decreased circulating leptin levels. Interestingly, this effect was not proportional to a decrease in fat mass but rather to direct effects of retinoic acid on leptin gene expression. Additionally, leptin expression has been reported to be increased in the BAT of mice fed a vitamin A deficient diet [90]. Retinoic acid is also able to decrease RBP4 secretion by 3T3-L1 and MEF cells [76]. Furthermore, subcutaneous injections of retinoic acid into lean mice led to a decrease in expression of WAT but not liver RBP4 expression, which was paralleled by improved insulin sensitivity in retinoic acid-treated animals [76]. 

## 4. Vitamin D

**Figure 2:**
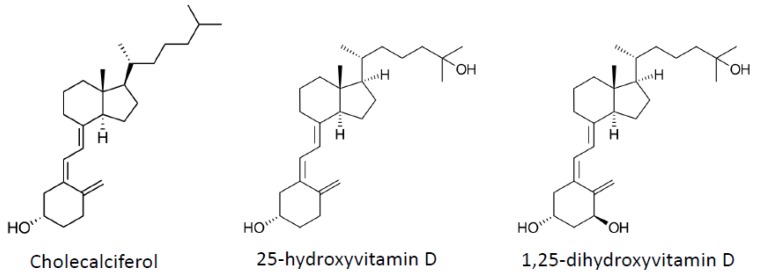
Vitamin D and metabolite structures.

Vitamin D (Figure 2) is a fat-soluble steroid hormone produced mainly in the skin upon exposure to ultraviolet B radiation, but that can also be supplied by the diet. To become biologically active, vitamin D must first be hydroxylated at position 25 to 25-hydroxyvitamin D (25(OH)D), which occurs mainly in the liver. This form is the main circulating form of vitamin D but is still biologically inactive. The active form is produced primarily in the proximal tubule of the kidney by hydroxylation at position 1 to obtain 1,25-dihydroxyvitamin D (1,25(OH)_2_D). 1,25(OH)_2_D is then released into the circulation by binding to a specific binding protein (Vitamin D Binding Protein), which is a carrier protein in the plasma. Locally, the prohormone can be converted to the bioactive hormone by the relative rates of cellular 1,25(OH)_2_D synthesis via CYP27B1 (1-hydroxylase) and hormone breakdown via CYP24 (24-hydroxylase) [93]. 

1,25(OH)_2_D mediates its biological effects by binding to the vitamin D receptor (VDR). The VDR belongs to the nuclear receptor superfamily of steroid/thyroid hormone receptors, and VDR is expressed by cells in most organs, including the brain, heart, skin, gonads, prostate and adipose tissue. The binding of 1,25(OH)_2_D to VDR allows it to act as a transcription factor. Indeed, the ligand-activated vitamin D receptor forms a heterodimer with RXR, which can bind to vitamin D response elements in various genes and cause the transactivation or repression of vitamin D-responsive genes [94]. 

Adipose tissue is considered the major reservoir for vitamin D even if data remain scarce [95]. In rats, it has been established that fat is the main storage site for vitamin D, where half of it is stored as non-metabolized vitamin D and the other half as polar metabolites, vitamin D esters, and unidentified compounds [96]. In obese patients, vitamin D was measured in subcutaneous adipose tissue [97] and visceral adipose tissue [98] confirming that adipose tissue is also a vitamin D reservoir in humans. Moreover, a strong correlation between adipose tissue vitamin D and serum vitamin D concentrations was found. Additionally, several studies reported an increase in 25(OH)D in plasma of obese people after bariatric surgery, suggesting that the reduction of adipose tissue mass could lead to release of vitamin D sequestered within adipose tissue [99]. However, this notion of sequestration has recently been challenged by Drincic *et al*., who showed that dilution of vitamin D in the large fat mass of obese patients fully explained their low vitamin D status [100]. 

In addition, the metabolism of vitamin D could be influenced by obesity. Indeed, several cytochrome p450 enzymes are modulated in obese women compared to lean women, resulting in increased catabolism and decreases in both 25- and 1α-hydroxylation [101]. These data indicated that, in addition to volumetric dilution [100], the metabolism of vitamin D was modified in the fat of obese people.

### 4.1. Adipogenesis

Several studies have explored the role of 1,25(OH)_2_D, the bioactive form of vitamin D, in differentiation and adipocyte metabolism [102]. Low concentrations of 1,25(OH)_2_D inhibited adipogenesis and reduced the accumulation of triacylglycerol. In addition, treatment of preadipocytes with other vitamin D metabolites, such as 24,25-dihydroxyvitamin D, also inhibited preadipocyte differentiation, but at higher concentrations than 1,25(OH)_2_D due to their low affinity for the VDR. One of the early effects of 1,25(OH)_2_D treatment of 3T3-L1 preadipocytes was an increase in VDR mRNA expression in preadipocytes [103], suggesting that the role of vitamin D in adipogenesis is VDR-dependent. These early studies also showed that specific 1,25(OH)_2_D binding was evident in preadipocyte 3T3-L1 cells but not in mature adipocytes [104]. Several years ago, Hida *et al.* showed that treatment of 3T3-L1 cells with 1,25(OH)_2_D inhibited adipogenesis in the presence of thiazolidinedione, which is a specific ligand for PPARγ, the master regulator of adipogenesis, and acts as a strong inducer of terminal differentiation in preadipocytes [105]. 

Two recent studies have focused on preadipocytes. The first study showed that 1,25(OH)_2_D inhibited porcine preadipocyte proliferation in a dose-dependent manner [106]. This inhibition might have been caused by induction of apoptosis by 1,25(OH)_2_D treatment. In this study, 1,25(OH)_2_D was shown not only to inhibit cell proliferation but also to block the differentiation of preadipocytes. These results indicate that 1,25(OH)_2_D plays a pivotal role in the inhibition of adipocyte differentiation. This effect could be due to suppression of transcription factors PPARγ and RXRα, which further down-regulates adipogenesis-related gene (*i.e.*, lipoprotein lipase, stearoyl-CoA desaturase 1, phosphoenolpyruvate carboxykinase, glycerol-3-phosphate dehydrogenase, and Glut4) expression [106]. Moreover, high doses of 1,25(OH)_2_D stimulated adipocyte apoptosis [107]. The second study performed by Kong and Li [108] confirmed that 1,25(OH)_2_D treatment inhibits adipocyte differentiation in 3T3-L1 preadipocytes, and they observed that the normal induction of a number of genes involved with the early stages of adipocyte development were affected in a dose-dependent manner by 1,25(OH)_2_D. In addition, they observed that removal of 1,25(OH)_2_D after 3 days of treatment allowed the differentiation process to be reinitiated. This important observation suggested that the main locus of the vitamin D effect on adipogenesis must reside in the suppression of a key reversible molecular event very early in the preadipocyte differentiation process. To conclude, this study suggests that 1,25(OH)_2_D inhibits adipogenesis in the 3T3-L1 cell model by likely suppressing CEBPα and PPARγ expression, antagonizing PPARγ transacting activity, and stabilizing VDR.

### 4.2. Inflammation

Several studies performed by Zemel and colleagues on 3T3-L1 and human adipocytes demonstrated that 1,25(OH)_2_D increased inflammatory cytokine expression and inhibited anti-inflammatory cytokine expression in both types of cells [109,110,111]. Accordingly, suppression of 1,25(OH)_2_D inhibited adipocyte-derived inflammation associated with obesity. However, recent studies have demonstrated a role completely opposite for 1,25(OH)_2_D_._ In fact, one study focused on the effect of 1,25(OH)_2_D on the production of proinflammatory chemokines/cytokines by human preadipocytes. 1,25(OH)_2_D significantly decreased the release of MCP-1, IL-8 and IL-6 from preadipocytes [112]. All these results suggested that vitamin D_3_ might protect against adipose tissue inflammation in obesity by lowering the release of MCP-1 and other proinflammatory cytokines from preadipocytes and disrupting the vicious cycle of macrophage recruitment. Another recent study showed that 1,25(OH)_2_D was able to attenuate adipose tissue inflammation by reducing MCP-1 expression [113]. In human mature adipocytes, 1,25(OH)_2_D decreased inflammatory cytokine IL-6 production and attenuated inflammation via NFκB protein nuclear translocation into the nucleus [114]. Very recently, we reported an anti-inflammatory role for 1,25(OH)_2_D in murine and human adipocytes. We further showed that 1,25(OH)_2_D decreased inflammatory marker expression. This anti-inflammatory effect is accompanied by increased glucose uptake by adipocytes. Therefore, the molecular mechanisms of regulation were unveiled and the implications of VDR and classical inflammation pathways, such as NF-κB or p38 MAP kinase, were confirmed [115].

### 4.3. Metabolism

It is well known that VDR has numerous activities, including the regulation of adipocyte biology and metabolism. In VDR^−/−^ mouse models, it was shown that VDR was implicated in the regulation of global energy metabolism *in vivo* [116,117]. Indeed, these mice were resistant to high fat diet-induced obesity. To determine more precisely the role of VDR in adipose tissue, the overexpression of human VDR in adipose tissue has been studied [118]. This overexpression led transgenic mice to obesity with increased body weight and fat mass, which was due to a decrease in energy expenditure, a reduction in fatty acid β oxidation and lipolysis. These effects are accompanied by the suppression of genes involved in these processes such as hexose kinase, carnitine palmitoyl transferase, hormone sensitive lipase, and adipose triglyceride lipase. In addition, the suppression of UCP1, UCP2 and UCP3 in transgenic mice also contributed to increased adipose mass. Altogether, these data showed that VDR was able to regulate global metabolism by exerting its effects on adipose tissue. It is noteworthy that CYP27B1^−/−^ mice, which do not synthesis 1,25(OH)_2_D are also lean, suggesting that not only VDR but also active vitamin D is involved in the regulation of energy expenditure, however the specific effect of a vitamin D supplementation is still unknown in this context. In addition the endogenous metabolism of vitamin D in adipose tissue remains unclear, which makes the interpretations of data generated in transgenic mice models complex and controversial. 

## 5. Vitamin E

**Figure 3:**
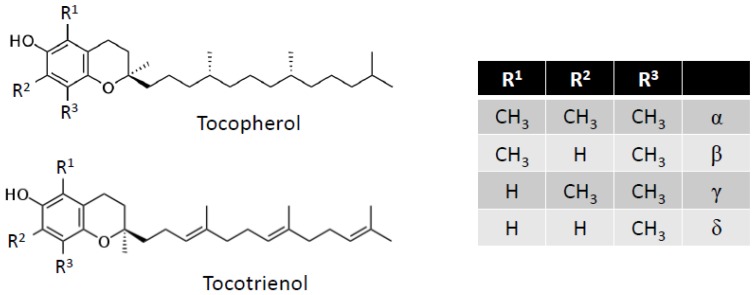
Vitamin E structure.

The generic term vitamin E (Figure 3) includes two major groups of molecules—tocopherols and tocotrienols—each with four vitamers: α, β, γ, and δ [119,120,121,122,123]. Their chemical structures consist of a mono-, di-, or tri-methylated chromanol ring attached to a 16-carbon atom side chain with an isoprene structure. This side chain defines the two major groups: Tocopherols have a saturated side chain and tocotrienols have a side chain bearing three unsaturated sites. The designation α, β, γ, or δ depends on the number and position of methyl groups on the aromatic ring. For the tocopherols, the existence of three asymmetric carbons (Position 2 on the chromanol ring and Positions 4′ and 8′ of the side chain) allows the existence of eight stereoisomers.

Absorbed vitamin E is found in different organs, but it is estimated that 90% of the total amount of vitamin E is stored in adipose tissue [124], specifically in the lipid droplets of adipocytes. The stromal-vascular fraction contains only a very small amount of vitamin E. The pool of vitamin E consists of about two-thirds α-tocopherol and one-third γ-tocopherol [125]. Tocotrienols are difficult to detect, but supplementation with tocotrienol in animals leads to increased concentrations of tocotrienols in adipose tissue [126]. 

The accumulation of tocotrienols (α- and γ-) is inhibited by α-tocopherol [127]. The plasma content of vitamin E can be strongly nutritionally modified over a few days; the stock in adipocytes, however, is much more stable and gives an indication of vitamin E intake over the long-term. Only a year-long supplementation can significantly increase the amount of vitamin E in adipose tissue [128]. This stability, which lasts several years, renders this stock poorly available when supplementation is stopped. However, it has recently been shown that the vitamin E present in adipose tissue can be mobilized very quickly (in a matter of a few weeks) under specific conditions of hypermetabolism, which can appear as a result of severe burns in children [129]. 

Beside its antioxidant effects, recent studies have shown that vitamin E is capable of modulating gene expression via a number of signaling pathways and nuclear receptors [122]. Indeed, it has been reported that α-and γ-tocotrienol and, to a lesser extent, α-and γ-tocopherol, are ligands of pregnane X receptor (PXR), a nuclear receptor involved in the metabolism of xenobiotics and in the catabolism of vitamin E [130]. It was also revealed that α-tocopherol acts specifically as an inhibitor of protein kinase C (PKC) activity via modulation of its degree of phosphorylation [131]. The α-tocopherol form is also capable of modulating the activation of transcription factors, such as nuclear factor kappa B (NF-κB) and activator protein-1 (AP-1) [132]. We have further shown that vitamin E is able to regulate the expression of genes dependent on the nuclear receptor PPARγ [133] and that α-tocopherol modulates the endogenous synthesis of cholesterol and oxysterols, likely by modulating the cleavage of SREBPs [134]. 

### 5.1. Adipogenesis

The effects of vitamin E on adipogenesis have recently been evaluated, and it appeared that the different vitamers do not have the same effects on adipocyte differentiation [135]. Whereas α-tocopherol seemed to have a stimulating effect on the expression of PPARγ and lipid accumulation during differentiation, tocotrienols (α and γ) inhibited the expression of PPARγ and a number of other markers of adipocyte differentiation. These effects resulted in a decrease in the accumulation of triglycerides. The decrease in phosphorylation of AKT in the presence of insulin could also be the cause of the observed inhibitory effects of tocotrienols.

### 5.2. Inflammation

The ability of γ-tocotrienol to limit the expression of inflammatory cytokines in response to TNFα stimulation has recently been described in adipocytes [136]. This anti-inflammatory effect was associated with an increase in adiponectin expression under the same conditions. Such an effect could be mediated via inhibition of the NF-κB pathway. This observation was in line with a previous study that reported an anti-inflammatory effect (decreased IL-6 and increased IL-10 expression in adipose tissue) of vitamin E (α-tocopherol) *in vivo* in mice fed a high fat diet, as well as in 3T3-L1 adipocytes stimulated by lipopolysaccharides (decreased IL-6) [137]. 

### 5.3. Metabolism

We examined the effect of α-tocopherol and γ-tocopherol on the expression of adiponectin. The induction of the latter, demonstrated in mice force-fed γ-tocopherol, was confirmed *in vitro* in a cellular model of 3T3-L1 adipocytes with not only γ-tocopherol but also α-tocopherol [133]. Given the critical role of PPARγ in the regulation of adiponectin, we examined the involvement of the nuclear receptor in this regulation using a specific antagonist of PPARγ that abolished the induction of adiponectin by tocopherols. Finally, we showed that tocopherols are not ligands of PPARγ but act via the nuclear receptor by modulating the amount of intracellular 15d prostaglandin J2 (15d PGJ2), a well-known PPARγ ligand. These data were later confirmed in an obese rat model (rats fed a high-fat diet) that showed increased synthesis of adiponectin by adipose tissue with vitamin E supplementation, resulting in increased plasma adiponectin [138]. Decreases in the adipose synthesis and plasma concentration of leptin were shown in this study, whereas an inductive effect of vitamin E on plasma leptin levels has been reported in humans [139]. The origin of this discrepancy remains unknown.

These studies showed that the accumulated data on gene regulation mediated by vitamin E in adipose tissue and/or adipocytes remain incomplete. However, the modulation of the expression of adiponectin or leptin, both of which are extensively involved in general homeostasis and energy metabolism along with PPARγ and its ligands, suggests broader effects with important metabolic consequences. In particular, we can easily speculate on the relationship of the anti-inflammatory effect of vitamin E with the well-established properties of PPARγ [140].

## 6. Vitamin K

**Figure 4:**
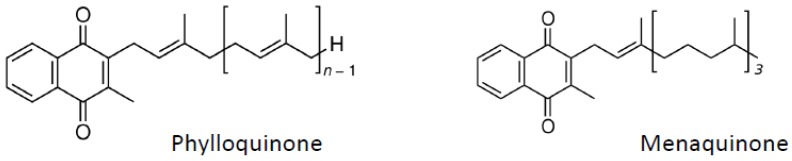
Vitamin K structure.

Vitamin K (Figure 4) occurs in various forms, including vitamin K1 (phylloquinone), which is present in plants, and vitamin K2 (menaquinones), which is synthesized by microorganisms and intestinal microbes. Vitamin K is involved in the carboxylation of several proteins. Thus this vitamin, considered a cofactor for the protein γ-carboxylation, may be involved in the regulation of blood coagulation, calcification, and energy metabolism or inflammation [141].

Vitamin K is also stored in adipose tissue, as recently demonstrated by Shea *et al.* [142]. No difference between subcutaneous and visceral fat was observed for storing vitamin K. Interestingly, vitamin K1 was the principal form of vitamin K detected in adipose tissue. This tissue was also an important reservoir of vitamin K in mice, but in mice, the concentration of vitamin K2 within fat was higher than that of vitamin K1 [143]. In addition to functioning as a cofactor in protein carboxylation, molecular effects of vitamin K are also notably mediated by pregnane X receptor (PXR), the xenobiotic receptor also activated by vitamin E [144].

The overall impact of vitamin K on adipose tissue/adipocytes is largely unknown. Little data are available regarding the anti-adipogenic effect of menaquinone on bone marrow stromal cells, which have the ability to differentiate into adipocytes under appropriate culture conditions [145,146]. No further data regarding the impact of vitamin K on adipocyte inflammatory status or metabolic impacts have been reported.

## 7. Carotenoids

**Figure 5:**
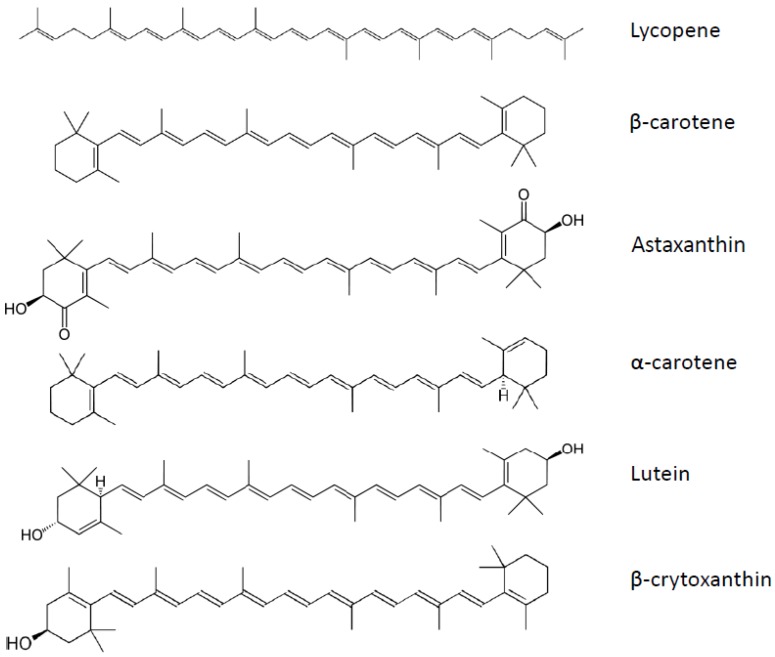
Carotenoids structure.

Carotenoids constitute a large family of more than 700 compounds. These pigments are present in variable quantities in fruits and vegetables. Among the carotenoids, there are six found predominantly in human plasma: β-carotene, α-carotene, lycopene, lutein/zeaxanthin, astaxanthin, and β-cryptoxanthin (Figure 5). The first three belong to the carotene sub-group, while the last three are xanthophylls. Epidemiological studies reported that consumption of a carotenoid-rich diet may be beneficial for human health [147]. Furthermore, some carotenoids can be cleaved by β-carotene 15,15′-monooxygenase (BCMO1) to release retinal, which is subsequently converted to retinol [148]. This group of carotenoids, which include β-carotene, α-carotene and β-cryptoxanthin, is called provitamin A. Carotenoids can also be cleaved by β-carotene 9′,10′-dioxygenase (BCDO2), leading to apo-carotenals [149].

Carotenoids are also stored in adipose tissue [150,151,152]. Adipose tissue concentrations of carotenoids are similar between men and women [152]. Lycopene was present at the highest concentration followed by β-carotene, and the total carotenoid concentration was the highest in the abdomen. Interestingly, most of the carotenoids appeared to be inversely correlated to fat mass, suggesting that during obesity carotenoids are sequestered in adipose tissue, decreasing their plasma concentrations. However, the concentration within adipose tissue is also lower in obese people [152]. Factors influencing distribution of carotenoids in adipose tissue are poorly understood, but we recently demonstrated that the uptake of carotenoid by adipose tissue was not linked to the carotenoid physicochemical properties [153]. Thus, the involvement of transporters was highly suspected. 

We also demonstrated the involvement of CD36 in lycopene and lutein uptake by adipose tissue and adipocytes. This study was conducted in vitro with siRNA or a specific inhibitor, as well as *ex vivo* using explant cultures of adipose tissues of CD36^−/−^ mice [154]. This was the first report on the involvement of a transporter in the uptake of carotenoids in adipose tissue. We also demonstrated that lycopene was mainly present within lipid droplets (approximately 50%), the rest being distributed between membranes (plasma and nuclear) in the cell [155]. Many others issues, such as the mechanisms of release of carotenoids from these adipose stores, deserve further investigation.

Molecular mechanisms mediating the effects of carotenoids on gene expression are varied. In the case of provitamin A carotenoids, RAR and RXR constitute specific signaling targets (presented in depth in the chapter dedicated to vitamin A), whereas other carotenoids, such as lycopene, regulate gene expression via ubiquitous signaling pathways such as NF-κB and MAP kinases [156]. Transcription factors involved in detoxification are also transactivated by carotenoids. This is the case for AhR, NRF2 or PXR [157,158]. In adipose tissue or adipocytes, however, no impact of carotenoids has been documented through these signaling pathways. 

### 7.1. Adipogenesis

The impact of some carotenoids has been documented in adipogenesis. Most of the reported effects inhibited adipocyte differentiation [159] by interfering with nuclear receptors such as RAR, RXR or PPAR. Indeed, β-carotene inhibited adipogenesis through the production of apo-carotenal (β-apo-14′-carotenal, but not β-apo-8′-carotenal) and repression of PPARα, PPARγ and RXR activation [160]. This was also the case of β-cryptoxanthin, which suppressed adipogenesis via activation of RAR [161], or astaxanthin, which inhibited rosiglitazone-induced adipocyte differentiation by antagonizing transcriptional activity of PPARγ [162]. Other carotenoids or metabolites did not modulate adipogenesis, as was the case with apo-10′-lycopenoic acid [163] and lycopene [164].

### 7.2. Inflammation

Anti-inflammatory effects of β-carotene in 3T3-L1 adipocytes were suggested to arise through limitation of TNFα-mediated down-regulation of genes linked to adipocyte biology [165]. The most studied anti-inflammatory carotenoid is lycopene, and we have demonstrated its ability to inhibit proinflammatory cytokine and chemokine expression *in vitro* (murine and human adipocytes) [56]. These data were also reproduced *ex vivo* on adipose tissue explants from mice subjected to a high fat diet (characterized by low-grade inflammation). The molecular mechanism was investigated and the involvement of NF-κB was confirmed. Similar results (*i.e.*, inhibition of cytokine and chemokine expression in various *in vitro* and *ex vivo* models) were obtained with apo-10′-lycopenoic acid, one metabolite of lycopene [163]. Finally, lycopene attenuated LPS-mediated induction of TNFα in macrophages via NF-κB and JNK [166], as well as macrophage migration *in vitro*. Consequently, lycopene decreased macrophage-induced cytokines, acute phase proteins and chemokine mRNA in adipocytes. Together these data suggested that lycopene is an anti-inflammatory compound active in adipocytes and macrophages, and can also reduce the vicious cycle between these two cellular types occurring in adipose tissue during low-grade inflammation associated with obesity. 

### 7.3. Metabolism

Astaxanthin prevented obesity in mice fed a high fat diet [167]. This effect was due to limited adipose tissue expansion. Similar anti-obesity effects have been documented in mice subjected to a high fat and high fructose diet [168] where insulin sensitivity and inflammation were also improved by astaxanthin. Anti-adiposity has also been reported for β-cryptoxanthin [169].

Effects for the lycopene metabolite apo-10′-lycopenoic acid on adipose metabolism have been suggested. Indeed, we reported that apo-10′-lycopenoic acid acted on RAR to modify adipocyte biology similarly to ATRA (see chapter on vitamin A) [163].

Significant research has been dedicated to the study of the impact of β-carotene on metabolism. The anti-obesity effect has subsequently been demonstrated to be linked to the provitaminic A effect [170,171] because BCMO1^−/−^ mice did not display adipose tissue weight modification. This effect was found to be linked to decreased expression of PPARγ in adipose tissue. Surprisingly, opposite results were obtained in ferret subcutaneous adipose tissue after β-carotene supplementation [172]. Finally, Lobo *et al.* confirmed the decrease of PPARγ expression upon β-carotene treatment and demonstrated the involvement of RAR signaling in this regulation [173].

## 8. Conclusion

Studies over the last 20 years have highlighted the beneficial effects of lipophilic micronutrients on several aspects of adipose tissue and/or adipocyte biology. The effects of vitamin A have been thoroughly investigated, but other vitamins and micronutrients have been far less studied. Therefore, significant work remains to obtain a global view of the effects of these molecules on adipose tissue biology. In addition several questions remain: How is the capture of LM permitted in adipocytes? What are the mechanisms of its intracellular trafficking, its storage in the lipid droplet, and its mobilization? 

Based on already acquired results, it appears that lipophilic micronutrients modulate several key processes occurring in adipocytes/adipose tissue via the regulation of gene expression. This modulation may explain the beneficial effects of these molecules in the context of obesity and associated pathologies, but several validations *in vivo* and in clinical studies will be necessary to support this concept.
